# Modulation of thyroidal radioiodide uptake by oncological pipeline inhibitors and Apigenin

**DOI:** 10.18632/oncotarget.5172

**Published:** 2015-09-09

**Authors:** Aparna Lakshmanan, Daniel Scarberry, Jill A. Green, Xiaoli Zhang, Samia Selmi-Ruby, Sissy M. Jhiang

**Affiliations:** ^1^ Department of Physiology and Cell Biology, The Ohio State University, Columbus, OH-43210, USA; ^2^ Molecular, Cellular and Developmental Biology Graduate Program, The Ohio State University, Columbus, OH-43210, USA; ^3^ Comprehensive Cancer Center, The Ohio State University, Columbus, OH-43210, USA; ^4^ Center for Biostatistics, The Ohio State University, Columbus, OH-43210, USA; ^5^ Centre de Recherche en Cancérologie de Lyon – UMR 1052- INSERM (Institut National de la Santé et de la Recherche Médicale, INSERM, Faculté de Médecine RTH Laennec, F-69372 Lyon, France

**Keywords:** thyroid cancer, NIS, TGF-β, apigenin, GDC-0941

## Abstract

Targeted radioiodine therapy for thyroid cancer is based on selective stimulation of Na^+^/I^−^ Symporter (NIS)-mediated radioactive iodide uptake (RAIU) in thyroid cells by thyrotropin. Patients with advanced thyroid cancer do not benefit from radioiodine therapy due to reduced or absent NIS expression. To identify inhibitors that can be readily translated into clinical care, we examined oncological pipeline inhibitors targeting Akt, MEK, PI3K, Hsp90 or BRAF in their ability to increase RAIU in thyroid cells expressing BRAF^V600E^ or RET/PTC3 oncogene. Our data showed that (1) PI3K inhibitor GDC-0941 outperformed other inhibitors in RAIU increase mainly by decreasing iodide efflux rate to a great extent; (2) RAIU increase by all inhibitors was extensively reduced by TGF-β, a cytokine secreted in the invasive fronts of thyroid cancers; (3) RAIU reduction by TGF-β was mainly mediated by NIS reduction and could be reversed by Apigenin, a plant-derived flavonoid; and (4) In the presence of TGF-β, GDC-0941 with Apigenin co-treatment had the highest RAIU level in both BRAF^V600E^ expressing cells and RET/PTC3 expressing cells. Taken together, Apigenin may serve as a dietary supplement along with small molecule inhibitors to improve radioiodine therapeutic efficacy on invasive tumor margins thereby minimizing future metastatic events.

## INTRODUCTION

Thyroid cancer incidence is rapidly increasing in the Unites States [1]. For thyroid cancer of poor prognosis, radioactive iodide (RAI) is administered for remnant ablation, and/or for targeted therapy of residual, recurrent, or metastatic cancer after total thyroidectomy. Targeted RAI therapy is facilitated by selective stimulation of Na^+^/I^−^ Symporter (NIS; SLC5A5)-mediated iodide uptake by thyrotropin (TSH) in thyroid cells. However, patients with refractory or advanced thyroid cancer could not benefit from RAI targeted therapy due to absent or insufficient TSH-stimulated RAI uptake (RAIU). Hence, much effort has been focused on identifying molecular mechanisms and reagents that could further increase TSH-stimulated thyroidal RAIU.

The BRAF^V600E^ oncogene is the most common mutation found in thyroid cancer followed by the RET/PTC rearrangements [2]. MEK/ERK pathway is a canonical downstream effector of BRAF^V600E^ while both MEK/ERK and PI3K/Akt pathways are well-accepted downstream arms of the activated tyrosine kinases resulting from RET/PTC rearrangements. Hsp90 is a chaperone that stabilizes client proteins including BRAF^V600E^ [3, 4] and RET/PTC [5] and maintains MEK and Akt signaling pathways [4, 6]. Pharmacological inhibitors targeting BRAF/MEK [7, 8], PI3K/Akt [8–10] or Hsp90 [5] selectively increase RAIU in thyroid cell cultures and their modes of action have been summarized [11]. The effects of inhibitors for BRAF/MEK [12] and Hsp90 [13] to increase RAIU have been validated in mouse models of thyroid cancer. Recently, a MEK inhibitor AZD6244 and a BRAF^V600E^ inhibitor GSK2118436 were reported to further increase thyroidal RAI accumulation to an extent that may lead to clinical benefit in RAI-refractory patients in a Phase 2 clinical trial [14] and in a pilot study [15] respectively.

To identify inhibitors that can be readily translated into clinical care, we investigated and compared several small molecule inhibitors (abbreviated as ‘i’) in oncological pipelines targeting Akt, MEK, PI3K, Hsp90 or BRAF in their ability to further increase TSH-stimulated RAIU in thyroid cells. None of the established human thyroid cell lines maintain TSH-stimulated NIS expression and RAIU. Thus, we chose PCCl3 rat thyroid cells that remain responsive to TSH in increasing NIS expression and RAIU for this study. To mimic thyroid tumors expressing BRAF^V600E^ or RET/PTC3 oncogenes, we examined PCCl3 cells with inducible expression of BRAF^V600E^ or RET/PTC3. TGF-β is a cytokine found in tumor microenvironment that has been shown to decrease NIS expression and thus RAIU at the invasive fronts of thyroid cancers [16–20]. We investigated if the effect of small molecule inhibitors on TSH-stimulated RAIU is compromised by TGF-β. Finally, we also examined the effect of Apigenin, a plant-based flavonoid, as a combination treatment with selected inhibitors as we previously reported that Apigenin further increased Akt inhibitor-induced RAIU [21].

## MATERIALS AND METHODS

### Cell culture and reagents

Immortalized PCCl3 rat thyroid cells were maintained in 6H media with 5% bovine serum as described earlier [10]. PCCl3 rat thyroid cells are authenticated as thyroid cells as they express NIS upon TSH stimulation. The absence of human cell line contamination is confirmed by western blots where NIS is detected only by rat NIS antibody and not detected by human NIS antibody. PCCl3 TetOn-BRAF^V600E^ cells [22] and PCCl3 TetOn-RET/PTC3 cells [23], generous gifts from Dr. James Fagin, Memorial Sloan Kettering Cancer Center, New York, NY, were genetically modified from PCCl3 cells to allow doxycycline (dox)-inducible expression of BRAF^V600E^ or RET/PTC3 oncogenes respectively. Schematic design of experiments is shown in Supplementary figure 1. Experiments were performed under acute TSH stimulation, where cells were withdrawn from TSH for 5 days (5H media) and then TSH was added back for 48 hours prior to treatment with various reagents for additional 24 hours. Reagents used in this study are listed as follows: MK-2206 (Akt1/2/3 inhibitor; Akti), GSK1120212, AZD6244 (MEK inhibitors; MEKi), GDC-0941, BKM-120 (PI3K inhibitors; PI3Ki), AUY-922 (Hsp90 inhibitor; Hsp90i), GSK2118436, PLX-4032 (BRAFV600E inhibitors; BRAFi) (Selleck Chemicals, Houston, TX), STA-9090 (Hsp90i) (Synta Pharmaceuticals Corp., Lexington, MA), Apigenin, DMSO (Sigma-Aldrich, St. Louis, MO) and TGF-β (Peprotech Inc. Rockyhill, NJ, USA).

### Concentration profiling of inhibitors

Optimal concentration (C_opt_) of inhibitors, defined as the concentration that increases RAIU to the greatest extent, was determined by performing RAIU on cells treated with inhibitor concentrations ranging from nanomolar to micromolar range to a maximum of up to 50 μM. If the greatest increase in RAIU was observed at a much lower concentration within the tested range, concentration as high as 50 μM was not tested. The results are summarized in Supplementary Table 1.

### RAIU and iodide efflux assays

These assays were performed as previously described [21, 24]. Note that RAIU assay measures RAI accumulation within cells, which reflects the equilibrium between NIS-mediated RAI influx and non-NIS-mediated RAI efflux.

### Western blot analysis

Cells were lysed and subjected to gel electrophoresis and Western blot analysis as previously described [10]. In this study, 4–20% gradient Tris-Glycine SDS-PAGE gels (Bio-Rad Laboratories Inc., Hercules, CA) were used. NIS protein was detected using PA716 rNIS polyclonal antibody (provided by S.S-R.) at a dilution of 1:1500. Phospho-ERK (pERK), total ERK, phospho-Akt (pAkt), total Akt and BRAF were detected using 1:1000 dilution of antibodies from Cell Signaling Technology, Inc. (Cat. # 9101, 9126, 9271, 9272) and Santa Cruz Biotechnology, Inc. (Dallas, TX; Cat. # sc-5284) respectively. Horseradish peroxidase-conjugated anti-rabbit or anti-mouse IgG secondary antibodies were used accordingly. Equivalent protein loading among samples was monitored by probing for GAPDH (Cell Signaling Technology, Inc.; Cat. 2118). Densitometry analysis was performed using ImageJ software.

### RNA extraction and quantitative real-time PCR

Total RNA was extracted using the RNeasy Kit (Qiagen, Venlo, Limburg) and contaminating DNA was removed by on-column DNase I digestion according to manufacturer's protocol. One microgram of extracted RNA was used for First-Strand Synthesis reverse transcription reaction (Invitrogen, Waltham, MA) performed according to the manufacturer's instructions. Quantitative real-time PCR (RT-qPCR) was performed in a 25μl reaction mixture, containing cDNA template, iQ SYBR Green PCR MasterMix (Bio-Rad Laboratories Inc., Hercules, CA) and primers. Reaction mixtures were incubated at 50°C for 2 min followed by an initial activation at 95°C for 3 min, and then subjected to 40 PCR cycles of denaturation (95°C for 15 s), annealing and extension using ABI HT7900 instrument (Applied Biosystems, Waltham, MA) by the Nucleic Acids Shared Resource at the OSUCCC. For Ano-1 and CFTR, annealing was at 58°C for 30 s and extension was at 72°C for 45 s. For Pendrin, ClC-5 and GAPDH, annealing and extension were at 60°C for 1 min. The primers used are as follows: Ano-1 (F: 5′-GAAACGGAAGCAGATGAGAC-3′; R: 5′-GGCTTCATACTCTGCTCTGG-3′) [25]; CFTR (F: 5′-GCGATGCTTTGTCTGGAGATTC-3′; R: 5′-CCACTTGTAAAGGAGCAATCCATA-3′) [26]; Pendrin (F: 5′-ACCGAGTCAAGGAATGGCTAC-3′; R: 5′-GATGGGGAAAAAGGCAGAGTA-3′) and ClC-5 (F: 5′-CTTACGCCAATGGAGATCGTA GTGG-3′; R: 5′-TCTTGGTTTGCCATCTGCGCTA-3′) [27]. Representative data are presented as relative fold changes of the target mRNA normalized to GAPDH mRNA.

### Statistical analysis

All experiments had at least two independent trials with three replicates for each experimental group within each trial. For RAIU assay, all the data values were log10 transformed to reduce variance and skewness, and then linear mixed effects models were used to take account of the correlations among observations from the same trial. For RAI efflux assays, the data was first normalized to the total iodide within cells at time 0 and then the percent remaining iodide at each time point was analyzed using linear mixed effects model. For RT-qPCR data, linear mixed effects models were used for analysis along with the ΔΔCT method. From the model, all the pre-specified comparisons for each experiment were obtained and adjusted for multiple comparisons using a sequentially rejective Holm's method [28] to control for type I error at 0.05. An interaction between two treatments is claimed synergistic only if [(A+B)-A)] > (B-control) and the difference [(A+B)-A]-(B-control) is significant after controlling for multiple testing. An interaction between two treatments is claimed additive if (A+B) is significantly better than A or B alone, but the synergy didn't reach significance. SAS v9.2 software was used for analysis (SAS Institute, Inc., Cary, NC).

## RESULTS

### The PI3Ki, GDC-0941, outperforms other inhibitors in further increasing TSH-stimulated RAI accumulation in BRAF^V600E^ expressing PCCl3 cells

The optimal concentration (C_opt_) that increased RAIU to the greatest extent was determined for each selected oncological pipeline inhibitor in PCCl3 cells, BRAF^V600E^ expressing PCCl3 cells, and RET/PTC3 expressing PCCl3 cells (Supplementary Table 1). The C_opt_ of Akti and PI3Ki were the same regardless of BRAF^V600E^ or RET/PTC3 overexpression. RET/PTC3 expressing cells required the highest C_opt_ for MEKi, yet a lower C_opt_ for Hsp90i. While BRAF^V600E^ expressing cells required the lowest C_opt_ for BRAFi GSK2118436, RET/PTC3 expressing cells required the lowest C_opt_ for BRAFi PLX-4032.

**Figure 1:**
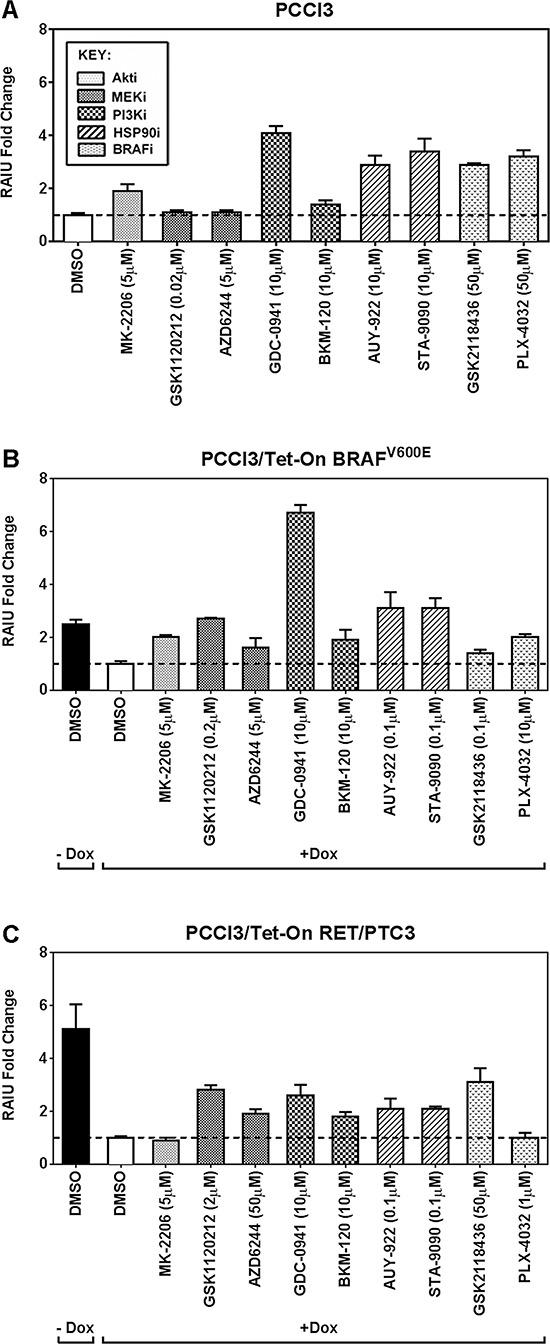
The PI3Ki, GDC-0941, outperforms other inhibitors in further increasing TSH-stimulated RAI accumulation in BRAF^V600E^ expressing PCCl3 cells The fold increase in RAIU by each inhibitor is shown in **A.** PCCl3 cells, **B.** PCCl3/Tet-On BRAF^V600E^ and **C.** PCCl3/Tet-On RET/PTC3 cells. Cells were deprived of TSH for five days and then stimulated with TSH for 48 hours, followed by treatment with inhibitors at their optimal concentration for 24 hours before RAIU analysis. For cells in (B) and (C), 2 μg/ml doxycycline (dox) was added with TSH to induce oncogene expression. Data are expressed as mean ± standard deviation (*n* = 3) and are representative of two independent trials.

The fold increase in RAIU by each inhibitor at its C_opt_ is shown in Figure 1. In PCCl3 cells, PI3Ki GDC-0941, Hsp90i and BRAFi significantly increased RAIU to a comparable extent of ∼3–4-fold (*p*-values < 0.01) (Figure 1A). In BRAF^V600E^ expressing PCCl3 cells, PI3Ki GDC-0941 outperforms the other inhibitors by significantly increasing RAIU to ∼7-fold (*p*-values < 0.0001) (Figure 1B). In RET/PTC3 expressing PCCl3 cells, MEKi GSK1120212, PI3Ki GDC-0941, and BRAFi GSK2118436 significantly increased RAIU to a comparable extent of ∼3-fold (*p*-values < 0.0001) (Figure 1C).

MEKi did not increase RAIU in PCCl3 cells but increased RAIU in BRAF^V600E^ or RET/PTC3 expressing cells, in which MEK pathway is overly activated. PI3Ki GDC-0941 and Hsp90i increased RAIU in PCCl3 cells and BRAF^V600E^ expressing cells to a greater extent than in RET/PTC3 expressing cells. Surprisingly, BRAFi only moderately increased RAIU in BRAF^V600E^ expressing cells. Taken together, our data indicate that PI3Ki GDC-0941 may be effective in further increasing TSH-stimulated RAI accumulation in BRAF^V600E^ expressing thyroid cancer cells as well as thyroid remnants.

### TGF-β reduces the extent of increase in TSH-stimulated RAIU by inhibitors

**Figure 2:**
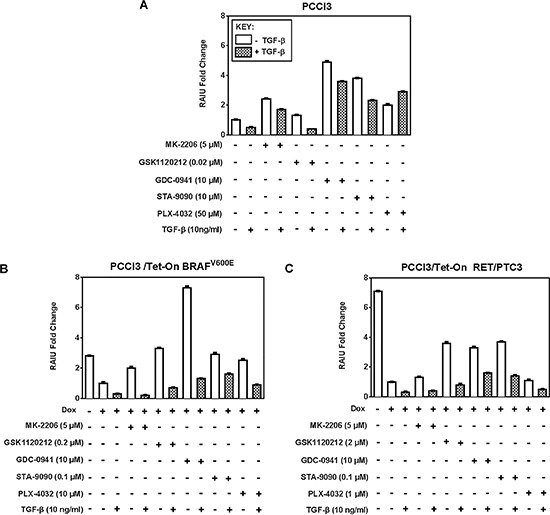
TGF-β reduces the extent of increase in TSH-stimulated RAIU by inhibitors Fold change of RAIU by inhibitors on TSH-stimulated RAIU in the presence of TGF-β is shown in **A.** PCCl3 cells, **B.** PCCl3/Tet-On BRAF^V600E^ and **C.** PCCl3/Tet-On RET/PTC3 cells. Cells were deprived of TSH for five days and then stimulated with TSH for 48 hours, followed by treatment with inhibitors at their optimal concentration with or without 10 ng/ml TGF-β for 24 hours before RAIU analysis. For cells in (B) and (C), 2 μg/ml doxycycline (dox) was added with TSH to induce oncogene expression. Data are expressed as mean ± standard deviation (*n* = 3) and are representative of two independent trials.

TGF-β, a cytokine present in the thyroid tumor microenvironment, not only promotes tumor invasiveness [16, 17] but also decreases NIS expression and RAIU [16, 18–20]. Consequently, the invasive thyroid cancer cells might be less targeted by RAI therapy. We examined the effects of inhibitors on TSH-stimulated RAIU in the presence of TGF-β to recapitulate the effects of tumor microenvironment. As shown in Figure 2, the extent of increase in RAIU by all inhibitors was greatly reduced by TGF-β in both BRAF^V600E^ expressing cells and RET/PTC3 expressing cells.

The increase in RAIU by Akti MK-2206 was completely abolished by TGF-β in both BRAF^V600E^ and RET/PTC3 expressing cells, yet was only moderately reduced by TGF-β in PCCl3 cells. RAIU reduction by TGF-β in MEKi GSK1120212 treated cells was equally extensive in all three cells. RAIU reduction by TGF-β among PI3Ki GDC-0941 treated cells was most pronounced in BRAF^V600E^ expressing cells, yet its RAIU level was much higher than TGF-β(+)/GDC-0941(−) cells. Interestingly, TGF-β did not reduce but increased RAIU in BRAFi PLX-4032-treated PCCl3 cells. Taken together, the efficacy of inhibitors in increasing TSH-stimulated RAIU in the invasive fronts of thyroid cancer is most likely to be compromised by the presence of TGF-β in tumor microenvironment. In the presence of TGF-β, PI3Ki GDC-0941 or Hsp90i STA-9090 conferred to higher RAIU than other inhibitors in both BRAF^V600E^ and RET/PTC3 expressing cells.

### Apigenin counteracts the effect of TGF-β on RAIU reduction

We previously reported that Apigenin, a plant-derived flavonoid, further augmented the increase of TSH-stimulated RAIU by Akt inhibitors [21]. In the absence of TGF-β, Apigenin co-treatment further increased RAIU only in combination with the Akti MK-2206 (*p*-values < 0.0001) but not with other inhibitors in all three cells examined (Supplementary Figure 2). The extent of increase in RAIU by Apigenin co-treatment with Akti MK-2206 was synergistic in BRAF^V600E^ or RET/PTC3 expressing cells but was additive in parental PCCl3 cells.

**Figure 3:**
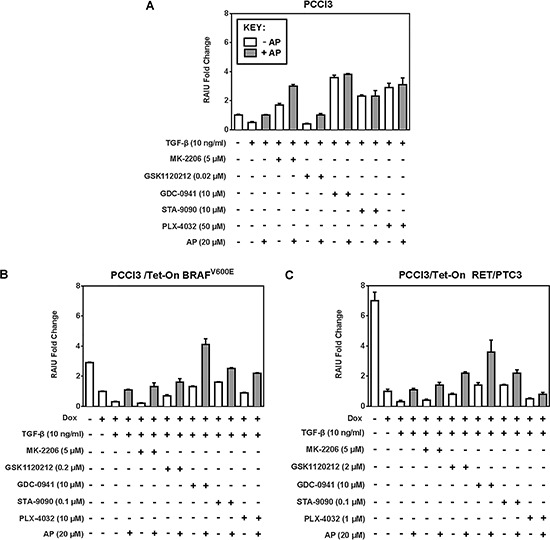
Apigenin counteracts RAIU reduction by TGF-β Fold change of RAIU by inhibitors on TSH-stimulated RAIU in the presence of TGF-β co-treated with Apigenin is shown in **A.** PCCl3 cells, **B.** PCCl3/Tet-On BRAF^V600E^ and **C.** PCCl3/Tet-On RET/PTC3 cells. Cells were deprived of TSH for five days and then stimulated with TSH for 48 hours, followed by treatment with inhibitors at their optimal concentration, co-treated with or without 20 μM of Apigenin (AP), in the presence of 10 ng/ml TGF-β for 24 hours before RAIU analysis. For cells in (B) and (C), 2 μg/ml doxycycline (dox) was added with TSH to induce oncogene expression. Data are expressed as mean ± standard deviation (*n* = 3) and are representative of two independent trials.

In PCCl3 cells, RAIU reduction by TGF-β was counteracted by Apigenin co-treatment in combination with Akti or MEKi, but not with PI3Ki or Hsp90i (Figure 3A). In BRAF^V600E^ expressing cells or RET/PTC3 expressing cells, RAIU reduction by TGF-β was counteracted by Apigenin co-treatment in combination with all the inhibitors examined (Figure 3B & 3C). Apigenin's counteraction to TGF-β's action on RAIU reduction was most evident in combination with Akti MK-2206, followed by PI3Ki GDC-0941 and MEKi GSK1120212 (*p*-values < 0.0001). Apigenin's effect was synergistic in combination with PI3Ki and additive in combination with MEKi and Hsp90i to increase RAIU. Apigenin's effect in increasing RAIU was neither synergistic nor additive to Akti MK-2206 as the increase of RAIU level by Apigenin alone was equivalent to Apigenin co-treatment with Akti MK-2206 in the presence of TGF-β.

In the absence of inhibitor treatment, TGF-β reduced RAIU and Apigenin reversed it in all three cells examined. The extent of RAIU reduction by TGF-β was greater in oncogene expressing PCCl3 cells than parental PCCl3 cells, i.e. 70% reduction versus 50% reduction. Similarly, the extent of reversing RAIU reduction by Apigenin was more evident in oncogene expressing PCCl3 cells than parental PCCl3 cells. Our data indicate that Apigenin may overcome RAIU reduction by TGF-β at the invasive fronts of thyroid cancer, in particular when Apigenin is administered in combination with PI3Ki, MEKi, or Hsp90i to further increase RAIU.

### Apigenin counteracts TGF-β's effect on NIS reduction

**Figure 4:**
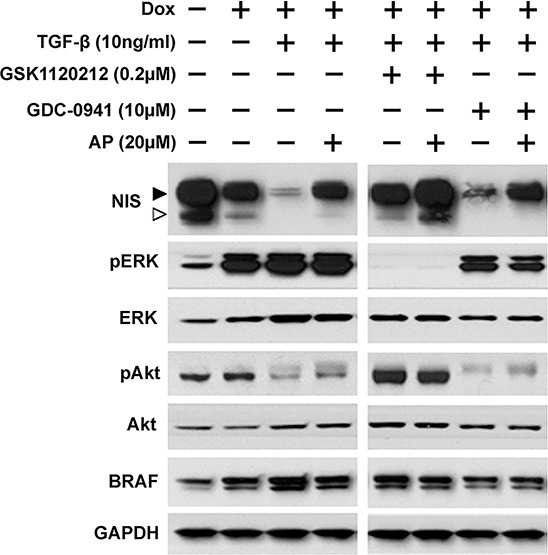
Apigenin counteracts TGF-β's effect on NIS reduction Western blots show NIS protein levels along with pERK, ERK, pAkt, Akt, and BRAF in PCCl3/Tet-On BRAF^V600E^ cells. Cells were deprived of TSH for five days and then stimulated with TSH for 48 hours, followed by treatment with inhibitors at their optimal concentration, co-treated with or without 20 μM of Apigenin (AP), in the presence of 10 ng/ml TGF-β for 24 hours before protein extraction. 2 μg/ml doxycycline (dox) was added with TSH to induce oncogene expression. GAPDH served as a loading control. Arrowheads indicate hyperglycosylated (►) and hypoglycosylated (▻) NIS. Data are representative of two independent trials.

Since BRAF^V600E^ oncogene is the most common mutation found in thyroid cancer and is associated with radioiodine refractory disease [29, 30], we examined NIS protein levels in BRAF^V600E^ expressing cells co-treated with TGF-β and inhibitors in the presence or absence of Apigenin. As shown in Figure 4, NIS protein level was decreased by dox induction of BRAF^V600E^, and TGF-β further decreased NIS protein level in BRAF^V600E^ expressing cells. NIS reduction by TGF-β was reversed by MEKi GSK1120212 but not by PI3Ki GDC-0941. However, Apigenin increased NIS protein levels in all TGF-β-treated cells with or without inhibitor treatment. TGF-β modestly increased pERK levels in BRAF^V600E^ expressing PCCl3 cells and Apigenin co-treatment had little effect on pERK levels. TGF-β extensively decreased pAkt level and Apigenin modestly increased pAkt level. Taken together, our data indicate that Apigenin reversed RAIU reduction in TGF-β treated cells (Figure 3) mainly by counteracting TGF-β's effect on NIS reduction (Figure 4).

### GDC-0941 decreases RAI efflux rate

**Figure 5:**
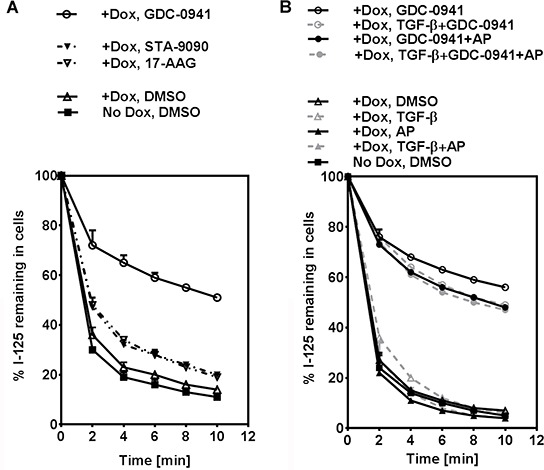
GDC-0941 decreases RAI efflux rate **A.** Iodide efflux rate in PCCl3/Tet-On BRAF^V600E^ cells treated with optimal concentration of PI3Ki GDC-0941, Hsp90i STA-9090 or Hsp90i 17-AAG is shown. **B.** Iodide efflux rate in PCCl3/Tet-On BRAF^V600E^ cells treated with optimal concentration of PI3Ki GDC-0941, co-treated with or without 20 μM of AP, in the presence or absence of 10 ng/ml TGF-β is shown. For (A) and (B), cells were deprived of TSH for five days and then stimulated with TSH for 48 hours, followed by treatment with reagents for 24 hours before efflux assay. 2 μg/ml doxycycline (dox) was added with TSH to induce oncogene expression. The iodide efflux rate is shown as the percentage of iodide remaining in the cells plotted at two-minute intervals. Data are representative of two independent trials.

We have previously reported that Hsp90i 17-AAG increases RAIU in thyroid cells at least in part by decreasing RAI efflux rate [5]. Since the extent of increase in RAIU by GDC-0941 was not accompanied by the same extent of increase in NIS protein levels or cell surface NIS levels (data not shown), we examined the effect of PI3Ki GDC-0941 on RAI efflux rate. GDC-0941 significantly decreased RAI efflux rate (*p*-values < 0.0001) to a much greater extent than Hsp90i STA-9090 or Hsp90i 17-AAG in BRAF^V600E^ expressing cells (Figure 5A) as well as PCCl3 cells (data not shown). As shown in Figure 5B, the decrease in RAI efflux rate by GDC-0941 was slightly reversed by co-treatment with TGF-β and/or Apigenin. However, TGF-β and Apigenin alone or in combination did not alter RAI efflux rate without co-treatment with GDC-0941. Taken together, GDC-0941 extensively decreased RAI efflux rate in BRAF^V600E^ expressing PCCl3 cells regardless of the presence of TGF-β or Apigenin.

### GDC-0941 may decrease RAI efflux rate in part by decreasing Pendrin and Ano-1 mRNA levels

Several transporters have been implicated in thyroidal iodide efflux, such as Pendrin (SLC26A4) [31–34], Anoctamin-1 (Ano-1; TMEM16A) [25, 35], Chloride Channel Voltage-sensitive 5 (ClC-5; CLCN5) [36] and Cystic Fibrosis Transmembrane Conductance Regulator (CFTR; ABCC7) [37]. To uncover the mechanisms underlying the decrease in RAI efflux rate by GDC-0941, we examined mRNA levels of these transporters in BRAF^V600E^ expressing cells treated with PI3Ki GDC-0941, Hsp90i STA-9090 or 17-AAG.

**Figure 6:**
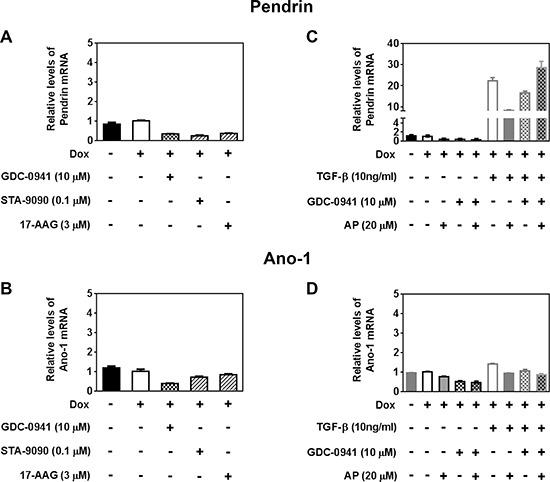
GDC-0941 may decrease RAI efflux rate in part by decreasing Pendrin and Ano-1 mRNA levels RT-qPCR results showing relative mRNA levels of **A, C.** Pendrin and **B, D.** Ano-1 in PCCl3/Tet-On BRAF^V600E^ cells treated with reagents as indicated. Cells were deprived of TSH for five days and then stimulated with TSH for 48 hours, followed by treatment with reagents for 24 hours before RNA extraction. 2 μg/ml doxycycline (dox) was added with TSH to induce oncogene expression. Data are expressed as mean ± standard deviation (*n* = 3) and are representative of two independent trials.

CFTR expression was not detected in these PCCl3 rat thyroid cells, in concordance with the observation that CFTR is not expressed in the FRT rat thyroid cell lines as well [38]. There was not much change in ClC-5 mRNA levels by the treatments (data not shown). Induction of BRAF^V600E^ expression did not alter the mRNA levels of these transporters. Pendrin mRNA level was decreased by Hsp90i STA-9090 and 17-AAG, which is consistent with previous report [39], as well as by PI3Ki GDC-0941 (Figure 6A). Ano-1 mRNA level was decreased by GDC-0941 to a greater extent than Hsp90i STA-9090 and 17-AAG (Figure 6B). Taken together, GDC-0941 may decrease RAI efflux rate in part by decreasing Pendrin and/or Ano-1 mRNA level.

We also examined whether co-treatment with TGF-β and/or Apigenin alters the levels of transporters in GDC-0941-treated cells. Apigenin co-treatment did not alter mRNA levels of Pendrin or Ano-1 in GDC-0941 treated cells (Figure 6C, 6D). TGF-β drastically increased Pendrin mRNA levels (Figure 6C) regardless of co-treatment with GDC-0941 and/or Apigenin. As shown in Figure 5B, co-treatment of TGF-β with GDC-0941 did increase RAIU efflux rate in the presence of BRAF^V600E^ induction. However, increased Pendrin mRNA level by TGF-β alone or in the presence of Apigenin did not translate into increased RAI efflux rate.

## DISCUSSION

In this study, we show that PI3Ki GDC-0941 outperforms other inhibitors in further increasing TSH-stimulated RAIU in BRAF^V600E^ expressing PCCl3 cells. We report that RAIU in the invasive fronts of thyroid cancer may be greatly reduced by TGF-β, even upon treatment with the inhibitors that were shown to increase TSH-stimulated RAIU in the absence of TGF-β. We found that RAIU reduction by TGF-β is mainly by decreasing NIS protein levels and NIS reduction by TGF-β can be counteracted by co-treatment of Apigenin. PI3Ki GDC-0941 had little effect on NIS protein levels but decreased iodide efflux rate to a great extent, thereby contributing to the increase in RAIU. Taken together, co-treatment of Apigenin with PI3Ki GDC-0941 may increase therapeutic efficacy of RAI for invasive fronts of thyroid cancer, by utilizing two distinct mechanisms—by increasing NIS protein level to counteract TGF-β's effect on NIS reduction and by decreasing RAI efflux rate, respectively.

The Akti MK-2206 increased RAIU in parental and BRAF^V600E^ oncogenic context but not in the RET/PTC3 context, suggesting that RAIU reduction in RET/PTC3 expressing PCCl3 cells is independent of Akt activation. MEKi GSK1120212 and AZD6244 increased RAIU in BRAF^V600E^ or RET/PTC3 expressing cells but not in parental PCCl3 cells, as MEK was not overly activated in parental PCCl3 cells. MEKi AZD6244 was reported to increase RAIU in 4/9, 5/5, 2/3 and 1/3 patients with BRAF^V600E^, NRAS, RET/PTC mutations and wild type respectively [14]. The BRAFi GSK2118436 has been shown to increase RAIU in 6/10 patients carrying BRAF^V600E^ mutation [15]. Both MEKi and BRAFi are known to increase RAIU mainly by increasing NIS protein level. In contrast, Akti and PI3Ki increase RAIU by increasing RAI influx rate [10] and by decreasing RAI efflux rate, respectively.

It is puzzling that PI3Ki GDC-0941 increased RAIU to a greater extent than MEKi in BRAF^V600E^ expressing cells, as MEK is the canonical downstream of BRAF^V600E^. One possible explanation is that MAPK activation by BRAF^V600E^ may also disturb the equilibrium of PI3K pathway, as cross-talk may occur between PI3K and MAPK at multiple levels [40]. As shown in Figure 4, MEKi (GSK1120212) abolished pERK level yet increased pAkt level; PI3Ki (GDC-0941) not only decreased pAkt level but also pERK level.

In thyroid cancers, TGF-β decreases NIS expression in the invasive margins [16, 17] that could enable the cells to escape RAI ablation and lead to RAI-refractory metastatic lesions. Our finding that Apigenin counteracts TGF-β's effect on RAIU reduction indicates that RAIU in invasive fronts of thyroid cancer can be increased to improve efficacy of RAI therapy. Apigenin has been reported to counteract the effects of TGF-β in other contexts as well, such as TGF-β-induced fibroblast to myofibroblast transition in human lungs [41] and TGF-β-induced VEGF expression in human prostate carcinoma cells by inhibiting Src/FAK/Akt and thereby Smad2/3 phosphorylation [42]. TGF-β in the tumor microenvironment is secreted both by the tumor cells and surrounding macrophages. While BRAF^V600E^ expressing cells secrete TGF-β in an autocrine loop [16], the addition of exogenous TGF-β recapitulates TGF-β secretion from macrophages. Apigenin has also been reported to reduce TGF-β production in human glioma cells [43], in rat renal cells damaged by cyclosporine [44], in rat mesangial cells induced by serotonin [45] and in human pancreatic stellate cells stimulated by parathyroid hormone-related protein [46]. Taken together, Apigenin may increase RAIU in the invasive fronts of thyroid cancer not only by counteracting TGF-β's action but also by decreasing TGF-β production from invasive tumor cells.

PI3Ki GDC-0941 had little effect on NIS protein levels but decreased iodide efflux rate to a great extent. PI3Ki BKM-120 had similar effect except the decrease in iodide efflux rate was much lesser than GDC-0941 (data not shown). These findings are different from studies showing that PI3Ki LY294002 increased NIS expression [9, 10] but had no effect on iodide efflux rate [9]. GDC-0941 is an ATP-pocket binding Class I PI3K inhibitor, structurally different from and more potent than BKM-120 [47]. GDC-0941 has fewer off-target effects even at micromolar concentrations compared to BKM-120 or LY294002 [47, 48]. One major difference between these Class I PI3K inhibitors is that GDC-0941 and BKM-120 target all four isoforms (p110 α/β/δ/γ) at nanomolar concentrations, while LY294002 targets only p110 α/β/δ but not p110γ even at micromolar concentrations. Thus, p110γ isoform may play a major role in modulating iodide efflux rate. Since Akti did not alter iodide efflux rate and pAkt level was low in TGF-β-treated BRAF^V600E^ expressing cells, the effect of GDC-0941 on decreasing iodide efflux rate is most likely mediated by other downstream effectors, such as PKCs, PKN, SGK or S6K [49].

Clinical trials are ongoing to confirm that RAI therapy can be improved by further enhancing TSH-stimulated RAIU with MEKi or BRAFi. However, it is concerning that RAIU increase by MEKi or BRAFi was considerably compromised by TGF-β, which is present in the invasive fronts of thyroid cancer. Our data showed that, in the presence of TGF-β, GDC-0941 with Apigenin co-treatment had the highest RAIU level in both BRAF^V600E^ expressing cells and RET/PTC3 expressing cells (Figure 3B, 3C). However, inherent differences in thyroid cells of different species [50, 51] are well recognized. Furthermore, the efficacy of small molecule inhibitors depends on the signaling context of cancer cells that may not be recapitulated in our oncogene-expressing PCCl3 cells. Thus, clinical translation of our findings requires further validation in preclinical mouse models as well as in human clinical trials. Once validated, Apigenin may serve as a dietary supplement along with small molecule inhibitors to counteract the effects of TGF-β on invasive tumor margins thereby minimizing future metastatic events.

## SUPPLEMENTARY MATERIAL FIGURES AND TABLE


