# Supplementary material for: Modulation of thyroidal radioiodide uptake by oncological pipeline inhibitors and Apigenin
Source: Oncotarget. 2015 Sep 9;6(31):31792–804. doi: 10.18632/oncotarget.5172 (PMC4741640; doi:10.18632/oncotarget.5172)
Supplement: Supplementary file 1 [file oncotarget-06-31792-s001.pdf]

SUPPLEMENTARY FIGURES AND TABLE

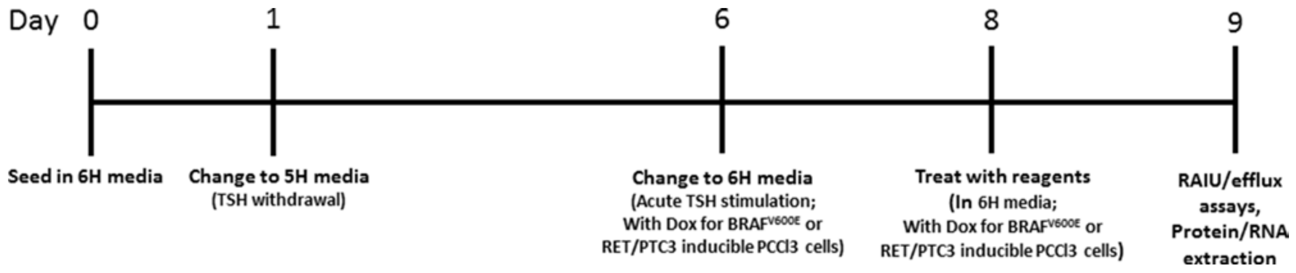

Supplementary Figure S1: Schematic design of experiments.

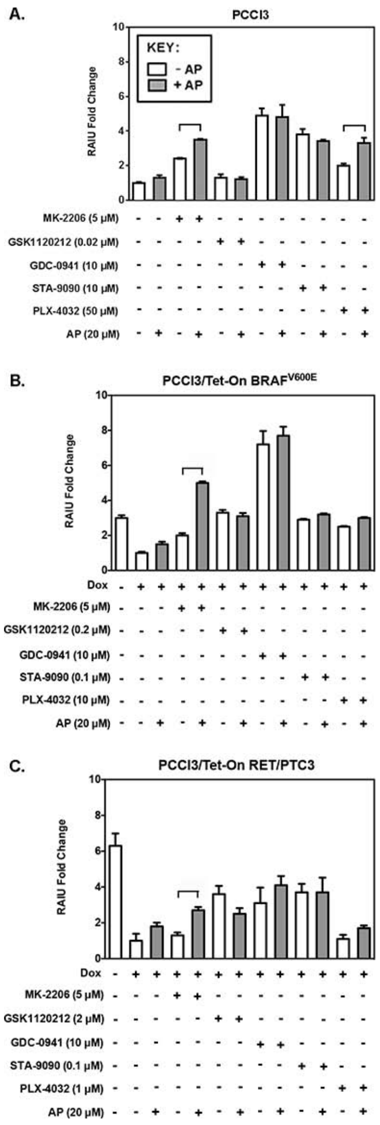

Supplementary Figure S2: Apigenin further augments the increase of TSH-stimulated RAIU by Akti MK-2206. Fold change of RAIU by inhibitors co-treated with Apigenin on TSH-stimulated RAIU is shown in **A.** PCCl3 cells, **B.** PCCl3/Tet-On BRAF^{V600E} and **C.** PCCl3/Tet-On RET/PTC3 cells. Cells were deprived of TSH for five days and then stimulated with TSH for 48 hours, followed by treatment with inhibitors at their optimal concentration with or without 20 µM of Apigenin (AP) for 24 hours before RAIU analysis. For cells in (B) and (C), 2 µg/ml doxycycline (dox) was added with TSH to induce oncogene expression. Data are expressed as mean ± standard deviation (*n* = 3) and are representative of two independent trials.

Supplementary Table S1: Optimal concentration of each inhibitor that increases RAIU in PCCl3 rat thyroid cells.

Signaling nodes targeted	Pipeline Inhibitors tested	PCCl3				PCCl3/Tet-On BRAF^{V600E}				PCCl3/Tet-On RET/PTC3			
		Concentration range tested (µM)	Optimal concentration (µM)	Fold increase in RAIU	Cytotoxic concentration (µM)	Concentration range tested (µM)	Optimal concentration (µM)	Fold increase in RAIU	Cytotoxic concentration (µM)	Concentration range tested (µM)	Optimal concentration (µM)	Fold increase in RAIU	Cytotoxic concentration (µM)
**Akt**	**MK-2206**	**0.005–50**	**5**	**2.0**	**50**	**0.005–50**	**5**	**2.0**	**50**	**0.005–50**	**5**	**1.0**	**50**
**MEK**	**GSK1120212 (Trametinib)**	**0.002–20**	**0.02**	**1.1**	**None**	**0.0002–2**	**0.2**	**2.6**	**None**	**0.002–20**	**2**	**2.8**	**None**
	AZD6244 (Selumetinib)	0.05–50	5	1.1	None	0.0005–50	5	1.6	None	0.05–50	50	1.9	None
**PI3K**	BKM120	0.01–50	10	1.2	None	0.01–50	10	1.7	None	0.01–50	10	1.9	None
	**GDC-0941**	**0.01–50**	**10**	**4.4**	**None**	**0.01–50**	**10**	**7.1**	**None**	**0.01–50**	**10**	**3.3**	**None**
**Hsp90**	**STA-9090 (Ganetespib)**	**0.1–50**	**10**	**3.4**	**None**	**0.1–10**	**0.1**	**2.8**	**None**	**0.01–10**	**0.1**	**2.6**	**None**
	AUY-922	0.1–50	10	3.1	None	0.1–10	0.1	2.7	None	0.01–10	0.1	2.2	None
**BRAF**	GSK2118436 (Dabrafenib)	0.001–50	50	2.5	None	0.001–10	0.1	1.2	None	0.01–50	50	3.1	None
	**PLX4032 (Vemurafenib)**	**0.001–50**	**50**	**3.3**	**None**	**0.001–50**	**10**	**1.8**	**None**	**0.01–50**	**1**	**1.3**	**None**

Inhibitors in bold: Chosen for further studies
